# Quantification of variability in trichome patterns

**DOI:** 10.3389/fpls.2014.00596

**Published:** 2014-11-13

**Authors:** Bettina Greese, Martin Hülskamp, Christian Fleck

**Affiliations:** ^1^Computational Biology and Biological Physics, Faculty for Theoretical Physics and Astronomy, Lund UniversityLund, Sweden; ^2^Molecular Cell Biology and Developmental Genetics, Biocenter, Botanical Institute, Cologne UniversityCologne, Germany; ^3^Laboratory for Systems and Synthetic Biology, Wageningen UniversityWageningen, Netherlands

**Keywords:** noise, cell-to-cell variability, pattern formation, spatial data analysis, plant development, trichome patterning

## Abstract

While pattern formation is studied in various areas of biology, little is known about the noise leading to variations between individual realizations of the pattern. One prominent example for *de novo* pattern formation in plants is the patterning of trichomes on Arabidopsis leaves, which involves genetic regulation and cell-to-cell communication. These processes are potentially variable due to, e.g., the abundance of cell components or environmental conditions. To elevate the understanding of regulatory processes underlying the pattern formation it is crucial to quantitatively analyze the variability in naturally occurring patterns. Here, we review recent approaches toward characterization of noise on trichome initiation. We present methods for the quantification of spatial patterns, which are the basis for data-driven mathematical modeling and enable the analysis of noise from different sources. Besides the insight gained on trichome formation, the examination of observed trichome patterns also shows that highly regulated biological processes can be substantially affected by variability.

## 1. Introduction

Mathematical modeling has been used to study various biological patterning processes, such as trichomes and root hairs (Savage et al., 2008; Benítez et al., 2011), cell sizes in sepals (Roeder et al., 2010), hair follicles (Sick et al., 2006), fruit fly development (Reeves et al., 2006), and other systems (Othmer et al., 2009; Peltier and Schaffer, 2010). It has only recently become more popular to investigate the variance or variability within a system and to discuss the consequences of noise (see Box 1) (Kærn et al., 2005; Swain and Longtin, 2006; Maheshri and O'Shea, 2007; Wilkinson, 2009; Sánchez et al., 2013). Moreover, an evaluation of the robustness (see Box 1) of a patterning system requires a quantification of the variations in its inputs and outputs (Reeves et al., 2006). Some studies have been published that focus on models with a stochastic (see Box 1) component, e.g., the stochastic Boolean network (see Box 1) model for root hairs (Savage et al., 2008) or floral morphogenesis (Alvarez-Buylla et al., 2008) or noise in the initiation of new organs in phyllotaxis (Mirabet et al., 2012). Others examine the effect of noise on patterning using stochastic differential equations (see Box 1) (Sagués et al., 2007). However, although a rich tradition exists in studying the effect of noise on pattern formation using abstract sets of equations, only few studies from developmental biology can be found where the effect of intracellular noise and/or cell-to-cell variability on a developing pattern or structure was systematically taken into account (Little et al., 2013). While advances in data acquisition and experimental manipulations increase the feasibility and popularity of noise-related studies in single cell organisms (Paldi, 2003; Kærn et al., 2005; Swain and Longtin, 2006; Sánchez et al., 2013), quantitative comparisons of spatial patterns and testable predictions from mathematical models are needed in order to assess the influence of various types of noise on a developing organism (Lander, 2011). In particular, it is desirable not only to qualitatively study simulation results that arise from various perturbations of the model, but also to quantitively compare these with experimentally observed patterns. As far as we are aware, the latter aspect has rarely been studied so far. It is important to note that the existence of cell-to-cell variability is not necessarily an outcome of stochasticity, but may be due to deterministic (see Box 1) regulatory processes upstream of the observed process (Snijder and Pelkmans, 2011). Whatever the source of the variability is, the pattern will be affected by it. In many studies, reaction-diffusion systems (see Box 1) are used to describe the pattern formation process (Gierer and Meinhardt, 1972; Meinhardt and Gierer, 1974; Koch and Meinhardt, 1994). These models require some stochasticity in the initial values to start the patterning. It is thought that this initial variability among cells in a tissue stems from a spontaneous fluctuation of the abundance of the proteins involved in the process. However, apart from this, variability is neglected and the equations themselves are deterministic. To explicitly study noise in patterning, it is necessary to not only consider stochastic initial conditions but also to include some other type of stochasticity such as spatially or temporally varying parameters (Page et al., 2005; Woolley et al., 2011).

Box 1 **Glossary Box**

**Noise:** In general, some kind of variability or variation in a given system can be described as noise, which can imply that it is unwanted (as in repeated measurements, for example). However, recent studies in biology find also situations where variability is neutral or even beneficial. Cellular noise originally refers to the variability in gene expression levels, but is also used for apparently random differences between neighboring cells.

**Robustness vs. sensitivity:** A system or method that does not adapt to some (small) change is called robust while one that reacts to change with some adaptation is called sensitive. In sensitivity analysis, the amount of adaptation of a model toward changes in parameter values is studied.

**Deterministic vs. stochastic system:** A system is deterministic when its state is completely determined for all times from the starting conditions. In contrast, a stochastic (or random) system, sometimes called stochastic process, contains some stochasticity and hence evolves into different states even for the same starting conditions.

**Boolean network model:** A variable that can only have values 1 or 0, typically meaning “on” and “off,” is called Boolean. A Boolean network is a system of equations where the time and the variable states are discrete (i.e., taking distinct, separate values, e.g., “points in time”).

**Stochastic differential equations:** In general, deterministic equations that contain a function of some continuous variables as well as the derivatives of these variables are called differential equations. Typical examples in biology are equations that contain concentrations as variables, molecular interactions as functions of these concentrations, and their rates of change over time and space as the derivatives. Different biological processes (e.g., production, degradation, binding) contribute as several terms (i.e., parts) of the equations. If one or more of the terms are stochastic processes, the system represents stochastic differential equations.

**Reaction-diffusion system:** A set of differential equations that describe reactions, e.g., molecular interactions, and diffusion, i.e., some form of spatial spread, is often called a reaction-diffusion model/system.

**Planar point pattern:** The spatial arrangement of points or objects (e.g., trichomes) in space is called a point pattern. If the space is the two-dimensional plane, i.e., a flat surface, it is called a planar point pattern.

**Quadrat:** In order to obtain spatially resolved counts of objects (e.g., trichomes) distributed on a surface (e.g., a leaf), this surface can be divided into smaller units. These are called quadrats in ecology and geography, and they are often squares. For a complete survey of the objects on the surface, quadrats are placed systematically in rows and columns and the objects are counted in each quadrat. These quadrat counts can then be statistically analyzed.

**Tessellation:** A planar space (i.e., a flat, two-dimensional surface) can be divided into smaller polygons (i.e., planar figures with straight sides) which cover the original plane without any overlap or gaps. This is called a tessellation or a tiling. Everyday examples for tessellations are brick walls or floor tiles.

In plants, the question whether the spatial distribution is random or ordered was first investigated for developing stomata (Sachs, 1974; Rasmussen, 1986; Croxdale, 2000). Stomata patterns are well suited for investigation because the patterns are two-dimensional and occur on the organ surface, which makes them readily accessible. Stomata are an example of a biological realization of a planar point pattern (see Box 1) (Larkin et al., 1997; Torii, 2012). Another prominent example for such a pattern from the plant kingdom are epidermal hairs, called trichomes, for which the regularity of the patterning process has been studied (Greese et al., 2012). The spatial distribution of trichomes is regulated by a genetic network and involves cell-to-cell communication. Trichome formation is promoted by three proteins that form an activating protein complex which can be inhibited by a fourth protein (Hülskamp, 2004; Digiuni et al., 2008). Because the inhibitor is mobile (i.e., non-cell autonomous), it effectively coordinates the patterning process between cells. In order to enable data-driven modeling for pattern formation, it is necessary to derive and evaluate models based on experimental data. To this end, statistical methods are needed which are suitable for the available type and amount of data and the studied system.

## 2. Quantitative characterization of noisy point patterns

The quantification of spatial variability is tightly related to the determination of the degree of regularity in a specific pattern. In other words, to be able to describe any kind of variability between two patterns, a suitable method to describe each pattern by itself is needed. Understanding the geometrical properties of a biological pattern helps to explore its functional role and its development (Galli-Resta et al., 1999). Moreover, appropriate statistical measures will be needed to analyze the effect of system perturbations (e.g., mutations), which will help together with mathematical models to elucidate the mechanistic role of the different components in a regulatory network. In the following, we first outline how planar point patterns arising in biology have been analyzed and then focus on the methods applied to trichome patterns.

One statistical method frequently used to assess a point pattern is the mean neighbor distance, which is compared to the mean neighbor distance of a completely random distribution (Clark and Evans, 1954). This was applied to stomata distribution where typically an ordered distribution was found (Miskin and Rasmussen, 1970; Croxdale, 2000). Because the next neighbor method is simple, it is easy to apply. However, all detailed information or spatial aspects of the pattern are lost. Therefore, more advanced methods were applied to analyze the stomata pattern (Martins et al., 2011). Trichome patterns have been examined through tests for deviation from randomness using quadrat counts (see Box 1) and their ratio of variance to mean (Smith and Watt, 1986), through analogous tests using nearest neighbor distances (Larkin et al., 1996), and through classification of mutant patterns based on cluster frequency and distance between trichomes (Schnittger et al., 1999). A variety of more advanced methods have been discussed and compared for spatial point patterns in general, e.g., (Boots (1986); Legendre and Fortin, 1989; Chiu, 2003). Different indices of dispersion that are based on distances and counts in quadrats have been used to compare plant patterns (Pielou, 1960; Goodall and West, 1979). Measures based on various graphs such as the Voronoi diagram and the minimal spanning tree have been used to analyze biological point patterns (Dussert et al., 1987; Wallet and Dussert, 1997) and geographical settlement patterns (Boots, 1986), and different structure indices as well as correlation functions have been applied to quantify forest structures (Pommerening, 2002). An extensive discussion of methods to analyze the spatial structure of ecological populations, illustrated on vegetation data, is given by Legendre and Fortin (1989), which includes correlograms, spectral analysis, periodograms, variograms, clustering, mapping, and testing for autocorrelation. Alternative approaches that characterize the geometry and topology of spatial patterns are Minkowski functionals, which have been applied to chemical reaction-diffusion systems (Mecke, 1996) and galaxy clusters (Kerscher et al., 1997). A popular method to analyze stochastic patterns is the structure function, which is the Fourier transform of the density correlation function, the spatial pendant of the power spectrum (Torquato, 2002). Peaks in the structure function denote regularity of the stochastic pattern. However, many of the well-known methods used in physics and astronomy require large domains and/or large sample sizes, which make them less suitable for the data typically available for biological systems. For instance, the sample size needed to obtain smooth statistics for the structure function grows exponentially with the noise in the pattern (Bastian, 2013). An estimate reveals that for the noise present in trichome patterning a sample size of the order of 10^4^ would be necessary (Bastian, 2013). Because one leaf with its trichome distribution represents one realization of the noisy patterning process, this is beyond the currently available amount of data. In general, the variety of strategies applied to investigate spatial structure illustrates that the choice of method is not straightforward and depends on the data and the question to be examined.

**Figure 1:**
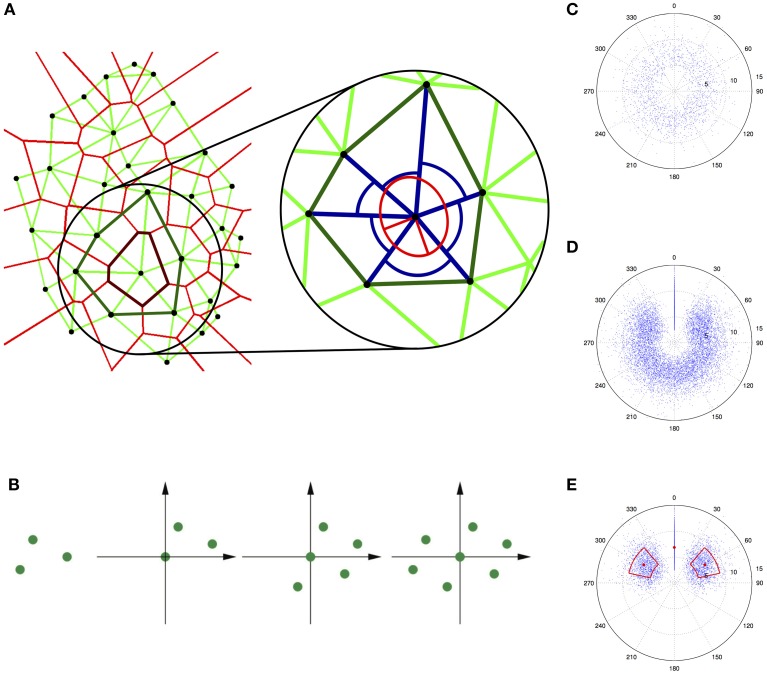
**Quantification of trichome patterns**. **(A)** Neighborhood measures for trichomes (black dots) on a single leaf. The left panel shows the Voronoi diagram (light red lines) and the modified Delaunay triangulation (light green lines) as well as the Voronoi polygon (dark red line) and the contiguous Voronoi polygon (dark green line) for a selected trichome. The right panel shows a magnification of one trichome (in the center) with its six neighbors and the neighbor distances and angles (blue lines and arcs). The anisotropy is related to the ratio of the principal axes of the ellipse (red lines). Reproduced with permisson from Greese et al. (2012) © The Institution of Engineering and Technology. **(B)** Construction of an autocorrelogram for a simple pattern containing three points. Three copies of the original pattern are superimposed such that each time one point lies in the origin of the coordinate system. **(C)** Truncated autocorrelogram for a data set with real trichome data. **(D)** Additionally rotated autocorrelogram. **(E)** Further reduced autocorrelogram where the mean and the standard deviation of the neighbor distances and angles are highlighted.

A suitable characterization of spatial trichome patterns is built on a tessellation (see Box 1) of the trichome positions, which splits the domain of the leaf into polygons that do not overlap or intersect, i.e., together they exactly cover the domain. A commonly used tessellation is the Voronoi diagram (Okabe et al., 2000), in which each point is assigned a polygon that contains that part of the domain that is closer to its defining point than to any other point (Figure 1A, left). Hence, the Voronoi diagram can be interpreted as an assignment of an influence area around each trichome that results from the inhibitory signal. Notably, the inverse of its area can be taken as a local density at its defining point (Duyckaerts et al., 1994). When pairs of trichomes whose Voronoi polygons share a common edge are connected by a straight line, the result is a Delaunay triangulation of the leaf (Figure 1A, left). The agglomerate of all triangles involving a selected trichome is called the contiguous Voronoi polygon, and it can also be used to calculate local density (Schaap and van de Weygaert, 2000). Various modifications have been proposed to adapt Delaunay triangulations to specific biological systems, resulting in different neighborhood graphs (Jaromczyk and Toussaint, 1992; Raymond et al., 1993). Pairs of neighbors can be defined by the edges present in the modified triangulation, such that each trichome is assigned a set of (mostly six) neighbors (Figure 1A, right) (Greese et al., 2012). Similar definitions of neighbors on graphs have been used elsewhere (Shapiro et al., 1985; Tanemura et al., 1991; Raymond et al., 1993; Duyckaerts et al., 1994; Eglen and Willshaw, 2002). For trichomes, the neighborhood concept has been used to restrict commonly used tessellation-based methods to the local scale that is important for developmental patterning systems (Greese, 2011; Greese et al., 2012).

A good visual impression of the order and symmetry inherent in a given point pattern can be obtained from a spatial autocorrelogram (Galli-Resta et al., 1999; Raven and Reese, 2002). This graphical representation of a given set of points is constructed by superimposing one copy of the pattern per point whereby the point is placed in the origin of the coordinate system (Figure 1B). For increasingly noisy patterns, the autocorrelogram becomes less distinct because the correlation is lost (first long-range, then short-range). If the region around the origin is devoid of points, i.e., an exclusion zone exists (Galli-Resta et al., 1999; Raven and Reese, 2002), the pattern exhibits a minimal distance between points, which can be seen as the simplest possible type of order (Larkin et al., 1996). The autocorrelogram can be used to extract the density recovery profile (Galli-Resta et al., 1999; Raven and Reese, 2002), which is an approximation of the autocorrelation function. In general, the central part of the autocorrelogram is most important for its interpretation, which allows a truncation of the plot to a chosen radius (compare Raven and Reese, 2002). The spatial autocorrelogram can be further adapted for the local analysis of trichome patterns and to avoid various artifacts (Figures 1C–E) (Greese, 2011). This modified autocorrelogram shows the distribution of neighbor distances and angles and hence gives a first impression about the regularity in a given point pattern. A more rigorous quantification that also allows easy comparison of different patterns requires the derivation of appropriate mathematical functions.

Measures suitable for the characterization of the regularity of the trichome pattern are local measures based on the neighborhood of each trichome. For each individual trichome, the distance to its neighbors, the angle between pairs of adjacent neighbors, and the anisotropy of the neighbors' distribution can be measured (Greese et al., 2012). The local anisotropy can be described using the eigenvalues of the inertia tensor (Goldstein et al., 2002). Their inverse values correspond to the length of the principal axes of an ellipse (Figure 1A, right), such that their ratio determines the deviation from isotropy (i.e., the case where the ellipse is a circle). For all measures it is advantageous to use the variation coefficient, i.e., the ratio of the standard deviation to the mean, in order to obtain measures independent of scale or density.

**Figure 2:**
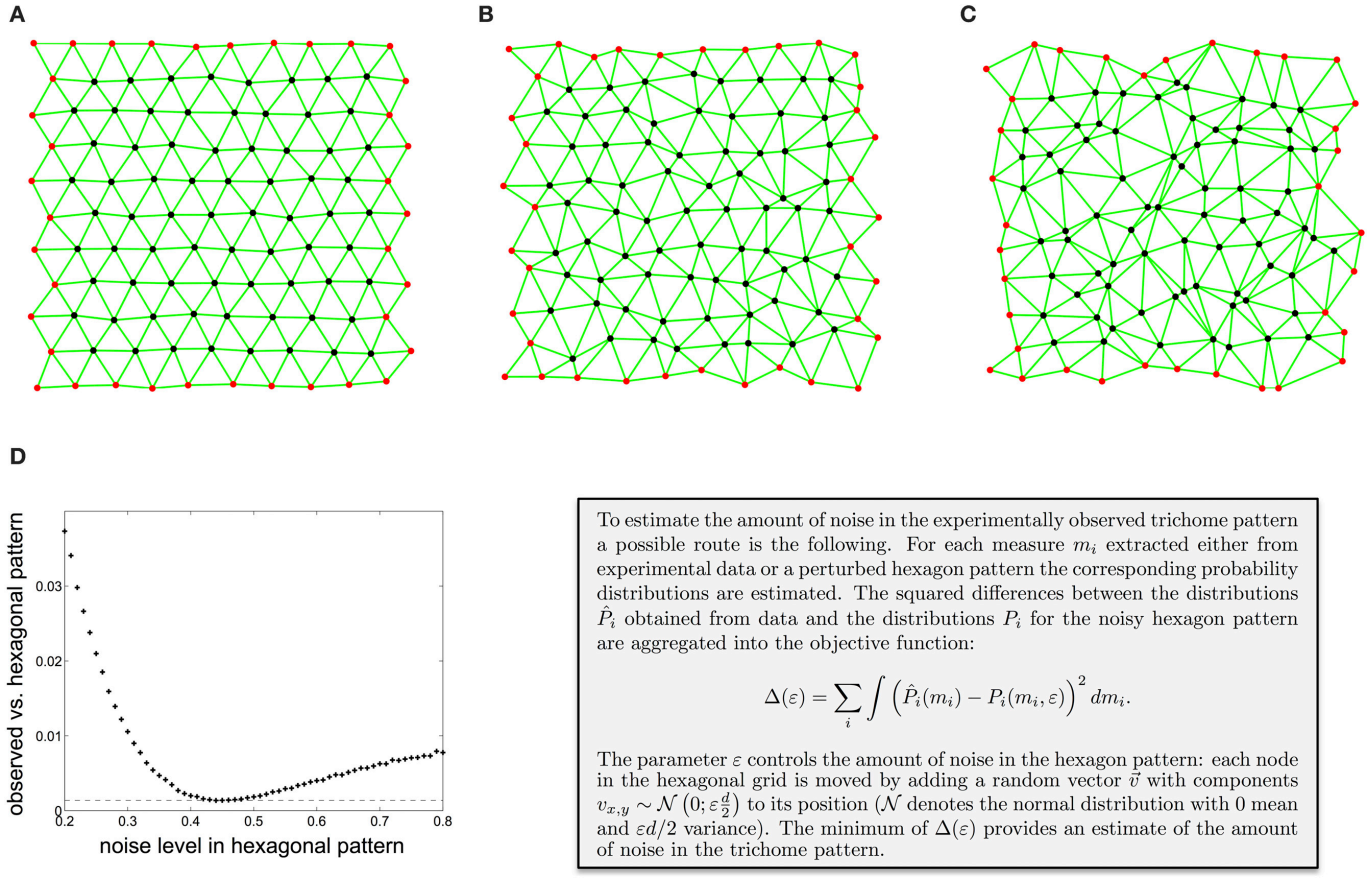
**Estimation of the amount of noise in the experimentally observed trichome pattern**. **(A–C)** A hexagon pattern with increasing amount of noise, controlled by the parameter ε (**A**: ε=0.1, **B**: ε=0.3, **C**: ε=0.5). **(D)** Difference between the local irregularity as measured by the distance between neighbors, the angle between pairs of adjacent neighbors, and the anisotropy of the neighbors distribution of the observed trichome and a noisy hexagonal pattern. The minimum shows that trichomes resemble a hexagonal pattern with a noise level of 0.44. Reproduced with permission from Greese et al. (2012) © The Institution of Engineering and Technology.

Other studies have used various related tessellation-based measures to characterize spatial point patterns (Boots, 1986; Marcelpoil and Usson, 1992; Duyckaerts et al., 1994; Croxdale, 2000; Schaap and van de Weygaert, 2000; Chiu, 2003), mostly focusing on the area of the Voronoi polygons or Delaunay triangles. Depending on whether one wants to detect differences in point density or measure spatial arrangement independent of point density, different measurements are appropriate. In order to estimate the overall amount of noise present in the observed trichome pattern, one can compare the values of the neighborhood measures obtained from experiments with the corresponding values for hexagonal point patterns with increasing noise level. Figures 2A–C show hexagon patterns for an increasing amount of irregularity (see text box for details). Aggregating the differences between the experimentally obtained values and the values for a noisy hexagonal point pattern into an objective function allows for estimation of the noise level which best reflects the noise in trichome patterning (Figure 2D, see Shapiro et al., 1985; Kinney et al., 2001 for similar calibration methods). This approach shows that trichomes show about 44% noise in relation to hexagonal patterns (see text box Figure 2 or Greese et al., 2012 for details), which is considerable for a tightly regulated patterning system. What does this mean for the patterning process? As it appears the patterning mechanism is important, as contrasted to a purely random process, to achieve a more or less homogeneous trichome density. The exact spatial distribution seems to be of less importance.

## 3. Sources of noise in trichome patterning

The trichome pattern is noisy, somewhat midway between a regular and a random pattern (Greese et al., 2012). But what are the sources of this observed irregularity? A clear distinction between different sources of noise (e.g., molecular processes, environmental conditions) is a challenge in any experimental or modeling study (Swain and Longtin, 2006). Because cells in a tissue will slightly vary in their protein content at a given time point (Kim and Price, 2010; Snijder and Pelkmans, 2011; Jeschke et al., 2013; Little et al., 2013), one first step is to investigate the effect of spatially varying initial states or reaction rates on the simulated trichome pattern (Page et al., 2005; Greese et al., 2012).

**Figure 3:**
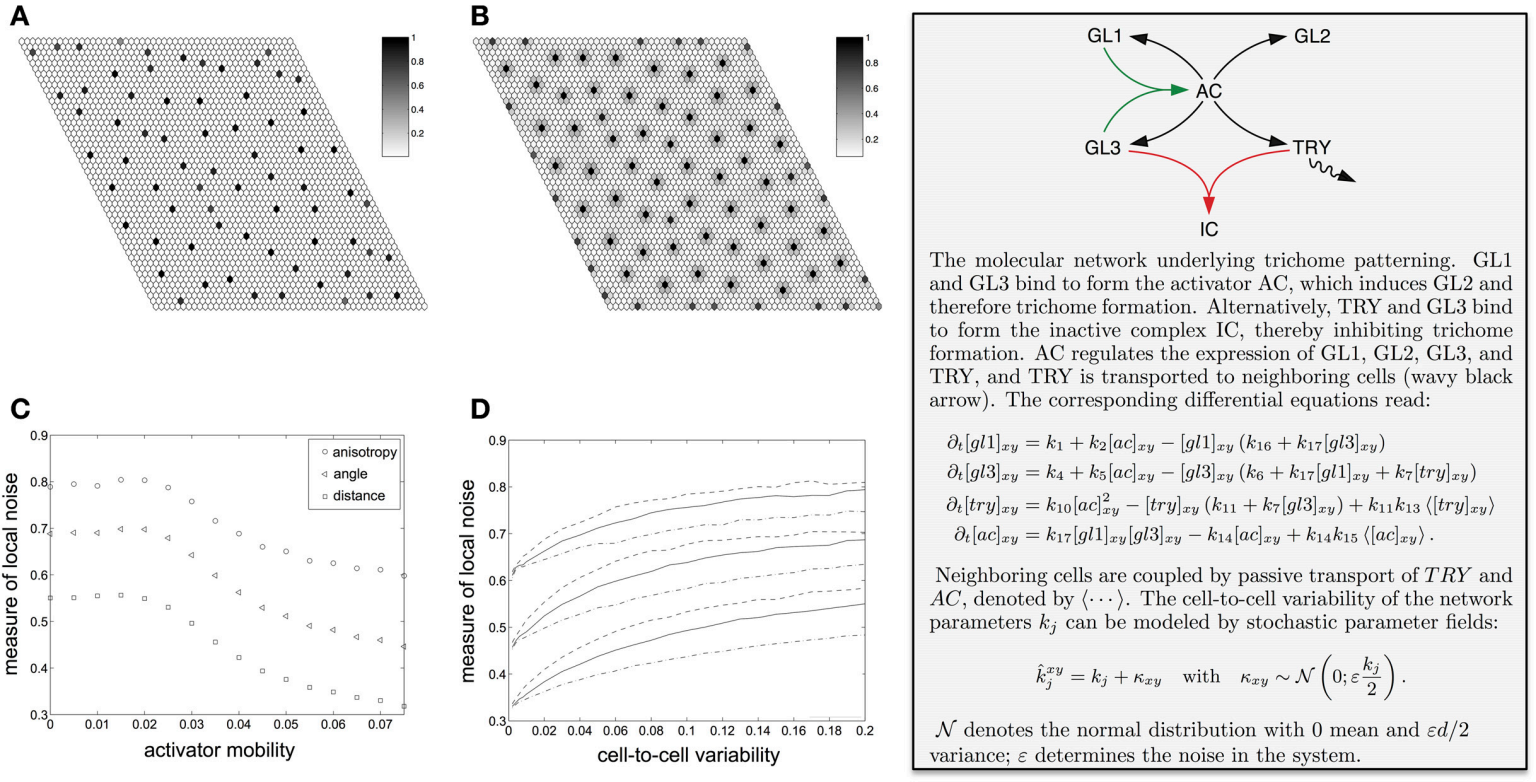
**Spatial variability in trichome patterns and influence of different sources of noise**. **(A,B)** Effect of the reduced activator mobility (*k*_15_). **(A)** Immobile activator (*k*_15_ = 0). This situation resembles the trichome patterning system as the activating complex of GL1 and GL3 is cell autonomous. The disorder from the random initial conditions remain in the final pattern. **(B)** With increasing activator mobility (*k*_15_ = 0.075) the peaks widen and the pattern becomes more regular. **(C)** Effect of noisy initial conditions on simulated trichome patterns with mobile activator. The plot shows the normalized mean variation coefficient of the neighbor distances (squares) and angles (triangles) as well as the normalized mean anisotropy (circles). All measures decrease for increasing activator mobility, thereby illustrating less variability. **(D)** Effect of random spatially inhomogeneous parameters on the simulated trichome pattern with mobile activator. The plot shows the mean relative neighbor measures (distances lower group, angles middle group, anisotropy upper group) for three selected model parameters that are represented by line styles. **(C,D)** All measures are normalized to the values of a random point pattern, i.e., zero denotes a perfectly regular and one a completely random point pattern. Reproduced with permission from Greese et al. (2012) © The Institution of Engineering and Technology.

The trichome initiation process resembles an activator-inhibitor system with an immobile activator (Gierer and Meinhardt, 1972; Meinhardt and Gierer, 1974; Koch and Meinhardt, 1994). If both, the activator and the inhibitor, are mobile, the resulting pattern depends only weakly on the initial conditions (Maini et al., 1997; Page et al., 2005). In a fast initial phase the early activator peaks are formed. These are usually not very pronounced. On a longer time-scale the activator peaks align and grow. Biologically, only the peaks at the later stage lead to an observable result, unless it becomes feasible to track the protein content in single cells in a tissue. The mobility of the activator allows the activator peaks to move slightly for optimal alignment (Holloway and Harrison, 1995; Ward and Wei, 2002). However, in the singular limit of vanishing activator mobility the optimal alignment of the peaks is impaired, and noise from the initial conditions remains in the final pattern. This can be seen in Figures 3A,B, where we show examples of simulation results for increasing mobility of the activator (see Figure 3, text box for further explanation). In Figure 3C the local irregularity of the simulated trichome pattern is plotted against the mobility of the activator (which is a complex consisting of GL1 and GL3 in case of the simulated trichome system). The pattern becomes more irregular with decreasing activator mobility, which is a known effect in reaction-diffusion systems (Holloway and Harrison, 1995). In other words, the cell autonomy of the activator in trichome patterning restricts the degree of regularity (see Greese et al., 2012 for details).

In general, if the parameters of a pattern formation network vary slightly from cell-to-cell, the resulting pattern will have a lower degree of regularity. How much the spatial variability of a single parameter affects the pattern depends on the details of the reaction network. Figure 3D shows as examples the dependence of the local irregularity on the activation of GLABRA3 (one of the patterning proteins) by the activator (solid lines), degradation of the activator (dashed lines), synthesis rate of GLABRA3 (dashed-dotted lines) (see Greese et al., 2012 for details). One interesting aspect of the effect of cell-to-cell variability on patterning is that protein binding rates strongly vary under conditions of macromolecular crowding (Minton, 2005, 2006; Grima, 2010). Because the abundance of molecules will, in general, slightly differ from cell to cell, the macromolecular crowding will vary and as a consequence also the protein-binding rates and in turn the resulting pattern will be less regular (Greese et al., 2012). The specific effects of crowding on gene expression have also been addressed in pure modeling and simulation studies, which were able to separate the effects related to binding and diffusion rates (Morelli et al., 2011) and to show the dependency on transcript levels on the volume fraction (Matsuda et al., 2014).

## 4. Perspective

To analyze spatial patterns in case of small sample sizes and few repetitions, it is useful to focus on methods that are sufficiently local and sensitive to subtle differences. Local measures which quantify the regularity of the local environment of each trichome as defined by tessellations can successfully be applied to compare experimental observation and results from computer simulations (Greese et al., 2012).

Any data analysis task poses several challenges, such as obtaining enough meaningful data, selecting appropriate methods, and linking observations to causes. The comparison of spatial patterns has often been done by simply looking at them and deciding whether they agree or not (sometimes referred to as “eye-balling”), which can be problematic as two realizations that look alike do not necessarily have to arise from the same mechanism. The use of simplistic measures may not be helpful as two very different patterns can lead to the same measured value, e.g., when the nearest neighbor distance is compared. Hence, it is important to carefully select the method(s) for analysis and make sure that the system under study can be adequately described and different situations can be discriminated. Regarding the analysis of noise, determining its overall magnitude is only the first step, the next—more interesting and challenging—part is then to trace it to various aspects of the system, in other words, find the sources of noise.

Variability is generally present in any natural process and hence introduces an additional level of complexity in the investigation of the process. A biological process may be strongly influenced or almost unchanged by noise, depending on the specific kind of variation and the properties of the particular system. Hence, the variability should—whenever possible—be treated as part of the process and not merely seen as a nuisance to be avoided. With the current trend of supplementing qualitative descriptions by quantitative measurements, it becomes not only feasible to estimate parameters for models but also necessary to compare results in more detail. In addition to a sound analysis of experimental data, it is very instructive to generate predictions with the help of a mathematical model because this approach allows the evaluation of different assumptions (including unrealistic situations like a noise-free system) and the separation of tightly coupled effects (like crowding on different reaction rates). All these efforts together will advance the understanding of the biological process under study.

### Conflict of interest statement

The authors declare that the research was conducted in the absence of any commercial or financial relationships that could be construed as a potential conflict of interest.

## References

[B1] Alvarez-Buylla E. R. Chaos Á Aldana M. Benítez M. Cortes-Poza Y. Espinosa-Soto C.. (2008). Floral morphogenesis: stochastic explorations of a gene network epigenetic landscape. PLoS ONE 3:e3626. 10.1371/journal.pone.0003626 18978941 PMC2572848

[B2] Bastian B. (2013). Spatial and Temporal Patterns in Biology. Diplomarbeit: Albert-Ludwigs-Universität Freiburg.

[B3] Benítez M. Monk N. A. M. Alvarez-Buylla E. R. (2011). Epidermal patterning in Arabidopsis: models make a difference. J. Exp. Zool. B Mol. Dev. Evol. 314B, 1–13. 10.1002/jez.b.21398 21259417

[B4] Boots B. N. (1986). Using angular properties of Delaunay triangles to evaluate point patterns. Geogr. Anal. 18, 252–260 10.1111/j.1538-4632.1986.tb00097.x

[B5] Chiu S. N. (2003). Spatial point pattern analysis by using Voronoi diagrams and Delaunay tessellations - a comparative study. Biometrical. J. 45, 367–376 10.1002/bimj.200390018

[B6] Clark P. J. Evans F. C. (1954). Distance to nearest neighbor as a measure of spatial relationships in populations. Ecology 35, 445–453 10.2307/1931034

[B7] Croxdale J. L. (2000). Stomatal patterning in angiosperms. Am. J. Bot. 87, 1069–1080. 10.2307/2656643 10947991

[B8] Digiuni S. Schellmann S. Geier F. Greese B. Pesch M. Wester K.. (2008). A competitive complex formation mechanism underlies trichome patterning on Arabidopsis leaves. Mol. Syst. Biol. 4:217. 10.1038/msb.2008.54 18766177 PMC2564731

[B9] Dussert C. Rasigni M. Palmari J. Rasigni G. Llebaria A. Marty F. (1987). Minimal spanning tree analysis of biological structures. J. Theor. Biol. 125, 317–323. 10.1016/S0022-5193(87)80063-2 3657213

[B10] Duyckaerts C. Godefroy G. Hauw J.-J. (1994). Evaluation of neuronal numerical density by Dirichlet tessellation. J. Neurosci. Methods 51, 47–69. 10.1016/0165-0270(94)90025-6 8189750

[B11] Eglen S. J. Willshaw D. J. (2002). Influence of cell fate mechanisms upon retinal mosaic formation: a modelling study. Development 129, 5399–5408. 10.1242/dev.00118 12403711

[B12] Galli-Resta L. Novelli E. Kryger Z. Jacobs G. (1999). Modelling the mosaic organization of rod and cone photoreceptors with a minimal-spacing rule. Eur. J. Neurosci. 11, 1461–1469. 10.1046/j.1460-9568.1999.00555.x 10103140

[B13] Gierer A. Meinhardt H. (1972). A theory of biological pattern formation. Kybernetik 12, 30–39. 10.1007/BF00289234 4663624

[B14] Goldstein H. Poole C. Safko J. (2002). Classical Mechanics, 3rd Edn. San Francisco, CA: Addison Wesley.

[B15] Goodall D. W. West N. E. (1979). A comparison of techniques for assessing dispersion patterns. Vegetatio 40, 15–27 10.1007/BF00052010

[B16] Greese B. (2011). Pattern Formation in Plant Developmental Biology: a Study of the Mechanisms Underlying Trichome Initiation Using Mathematical Modelling. Dr. rer. nat. Dissertation, Albert-Ludwigs-Universität Freiburg, Freiburg.

[B17] Greese B. Wester K. Bensch R. Ronneberger O. Timmer J. Hülskamp M.. (2012). Influence of cell-to-cell variability on spatial pattern formation. IET Syst. Biol. 6, 143–153. 10.1049/iet-syb.2011.0050 23039695

[B18] Grima R. (2010). Intrinsic biochemical noise in crowded intracellular conditions. J. Chem. Phys. 132:185102 10.1063/1.3427244

[B19] Holloway D. M. Harrison L. G. (1995). Order and localization in reaction-diffusion pattern. Physica A 222, 210–233 10.1016/0378-4371(95)00202-2

[B20] Hülskamp M. (2004). Plant trichomes: a model for cell differentiation. Nat. Rev. Mol. Cell Biol. 5, 471–480. 10.1038/nrm1404 15173826

[B21] Jaromczyk J. W. Toussaint G. T. (1992). Relative neighborhood graphs and their relatives. Proc. IEEE 80, 1502–1517 10.1109/5.163414

[B22] Jeschke M. Baumgärtner S. Legewie S. (2013). Determinants of Cell-to-Cell Variability in Protein Kinase Signaling. PLoS Comput. Biol. 9:e1003357. 10.1371/journal.pcbi.1003357 24339758 PMC3854479

[B23] Kærn M., Elston T. C. Blake W. J. Collins J. J. (2005). Stochasticity in gene expression: from theories to phenotypes. Nat. Rev. Genet. 6, 451–464. 10.1038/nrg1615 15883588

[B24] Kerscher M. Schmalzing J. Retzlaff J. Borgani S. Buchert T. Gottlöber S. (1997). Minkowski functionals of Abell/ACO clusters. Month. Notices R. Astron. Soc. 284, 73–84 10.1093/mnras/284.1.73

[B25] Kim P.-J. Price N. D. (2010). Macroscopic kinetic effect of cell-to-cell variation in biochemical reactions. Phys. Rev. Lett. 104:148103. 10.1103/PhysRevLett.104.148103 20481966

[B26] Kinney J. H. Oliveira J. Haupt D. L. Marshall G. W. Marshall S. J. (2001). The spatial arrangement of tubules in human dentin. J. Mater. Sci. Mater. Med. 12, 743–751. 10.1023/A:1011232912734 15348247

[B27] Koch A. J. Meinhardt H. (1994). Biological pattern formation: from basic mechanisms to complex structures. Rev. Mod. Phys. 66, 1481–1507 10.1103/RevModPhys.66.1481

[B28] Lander A. D. (2011). Pattern, growth, and control. Cell 144, 955–969. 10.1016/j.cell.2011.03.009 21414486 PMC3128888

[B29] Larkin J. C. Marks M. D. Nadeau J. Sack F. (1997). Epidermal cell fate and patterning in leaves. Plant Cell 9, 1109–1120. 10.1105/tpc.9.7.1109 9254933 PMC156984

[B30] Larkin J. C. Young N. Prigge M. Marks M. D. (1996). The control of trichome spacing and number in Arabidopsis. Development 122, 997–1005. 8631276 10.1242/dev.122.3.997

[B31] Legendre P. Fortin M. J. (1989). Spatial pattern and ecological analysis. Plant Ecol. 80, 107–138 10.1007/BF00048036

[B32] Little S. C. Tikhonov M. Gregor T. (2013). Precise developmental gene expression arises from globally stochastic transcriptional activity. Cell 154, 789–800. 10.1016/j.cell.2013.07.025 23953111 PMC3778922

[B33] Maheshri N. O'Shea E. K. (2007). Living with noisy genes: how cells function reliably with inherent variability in gene expression. Ann. Rev. Biophys. Biomol. Struct. 36, 413–434. 10.1146/annurev.biophys.36.040306.132705 17477840

[B34] Maini P. Painter K. Chau H. (1997). Spatial pattern formation in chemical and biological systems. J. Chem. Soc. Faraday Trans. 93, 3601–3610 10.1039/a702602a

[B35] Marcelpoil R. Usson Y. (1992). Methods for the study of cellular sociology: voronoi diagrams and parametrization of the spatial relationships. J. Theor. Biol. 154, 359–369 10.1016/S0022-5193(05)80176-6

[B36] Martins G. A. Soares A. M. Barbosa J. P. R. A. D. de Mello J. M. de Castro E. M. Ferraz A. C. (2011). Stomatal density distribution patterns in leaves of the Jatobá (*Hymenaea courbaril L*.). Trees 26, 571–579 10.1007/s00468-011-0620-4

[B37] Matsuda H. Putzel G. G. Backman V. Szleifer I. (2014). Macromolecular crowding as a regulator of gene transcription. Biophys. J. 106, 1801–1810. 10.1016/j.bpj.2014.02.019 24739179 PMC4008821

[B38] Mecke K. R. (1996). Morphological characterization of patterns in reaction-diffusion systems. Phys. Rev. E 53, 4794–4800. 10.1103/PhysRevE.53.4794 9964807

[B39] Meinhardt H. Gierer A. (1974). Applications of a theory of biological pattern formation based on lateral inhibition. J. Cell Sci. 15, 321–346. 4859215 10.1242/jcs.15.2.321

[B40] Minton A. P. (2005). Influence of macromolecular crowding upon the stability and state of association of proteins: predictions and observations. J. Pharm. Sci. 94, 1668–1675. 10.1002/jps.20417 15986476

[B41] Minton A. P. (2006). How can biochemical reactions within cells differ from those in test tubes? J. Cell Sci. 119(Pt 14), 2863–2869. 10.1242/jcs.03063 16825427

[B42] Mirabet V. Besnard F. Vernoux T. Boudaoud A. (2012). Noise and robustness in phyllotaxis. PLoS Comput. Biol. 8:e1002389. 10.1371/journal.pcbi.1002389 22359496 PMC3280957

[B43] Miskin K. E. Rasmussen D. C. (1970). Frequency and distribution of stomata in barley. Crop Sci. 10, 575–578. 10.2135/cropsci1970.0011183X001000050038x24212553

[B44] Morelli M. J. Allen R. J. ten Wolde P. R. (2011). Effects of macromolecular crowding on genetic networks. Biophys. J. 101, 2882–2891. 10.1016/j.bpj.2011.10.053 22208186 PMC3244068

[B45] Okabe A. Boots B. Sugihara K. Chiu S. N. (2000). Spatial Tessellations: Concepts and Applications of Voronoi Diagrams, 2nd Edn. Wiley Series in Probability and Mathematical Statistics. Chichester: Wiley 10.1002/9780470317013

[B46] Othmer H. G. Painter K. Umulis D. Xue C. (2009). The intersection of theory and application in elucidating pattern formation in developmental biology. Math. Model. Nat. Phenom. 4, 3–82. 10.1051/mmnp/20094401 19844610 PMC2763616

[B47] Page K. Maini P. Monk N. (2005). Complex pattern formation in reaction-diffusion systems with spatially varying parameters. Physica D 202, 95–115 10.1016/j.physd.2005.01.022

[B48] Paldi A. (2003). Stochastic gene expression during cell differentiation: order from disorder? Cell. Mol. Life Sci. 60, 1775–1778. 10.1007/s00018-003-23147-z 14523542 PMC11138758

[B49] Peltier J. Schaffer D. V. (2010). Systems biology approaches to understanding stem cell fate choice. IET Syst. Biol. 4, 1–11. 10.1049/iet-syb.2009.0011 20001088

[B50] Pielou E. C. (1960). A single mechanism to account for regular, random and aggregated populations. J. Ecol. 48, 575–584 10.2307/2257334

[B51] Pommerening A. (2002). Approaches to quantifying forest structures. Forestry 75, 305–324 10.1093/forestry/75.3.305

[B52] Rasmussen H. (1986). Pattern-formation and cell-interactions in epidermal development of anemarrhena-asphodeloides (Liliaceae). Nordic J. Bot. 6, 467–477 10.1111/j.1756-1051.1986.tb00903.x

[B53] Raven M. A. Reese B. E. (2002). Horizontal cell density and mosaic regularity in pigmented and albino mouse retina. J. Comp. Neurol. 454, 168–176. 10.1002/cne.10444 12412141

[B54] Raymond E. Raphael M. Grimaud M. Vincent L. Binet J. L. Meyer F. (1993). Germinal center analysis with the tools of mathematical morphology on graphs. Cytometry 14, 848–861. 10.1002/cyto.990140803 8287731

[B55] Reeves G. T. Muratov C. B. Schüpbach T. Shvartsman S. Y. (2006). Quantitative models of developmental pattern formation. Dev. Cell 11, 289–300. 10.1016/j.devcel.2006.08.006 16950121

[B56] Roeder A. H. K. Chickarmane V. Cunha A. Obara B. Manjunath B. S. Meyerowitz E. M. (2010). Variability in the control of cell division underlies sepal epidermal patterning in Arabidopsis thaliana. PLoS Biol. 8:e1000367. 10.1371/journal.pbio.1000367 20485493 PMC2867943

[B57] Sachs T. (1974). The developmental origin of stomata pattern in Crinum. Bot. Gazette 135, 314–318 10.1086/336767

[B58] Sagués F. Sancho J. García-Ojalvo J. (2007). Spatiotemporal order out of noise. Rev. Mod. Phys. 79, 829–882 10.1103/RevModPhys.79.829

[B59] Sánchez A. Choubey S. Kondev J. (2013). Regulation of noise in gene expression. Ann. Rev. Biophys. 42, 469–491. 10.1146/annurev-biophys-083012-130401 23527780

[B60] Savage N. S. Walker T. Wieckowski Y. Schiefelbein J. Dolan L. Monk N. A. M. (2008). A mutual support mechanism through intercellular movement of CAPRICE and GLABRA3 can pattern the Arabidopsis root epidermis. PLoS Biol. 6:e235. 10.1371/journal.pbio.0060235 18816165 PMC2553841

[B61] Schaap W. E. van de Weygaert R. (2000). Continuous fields and discrete samples: reconstruction through Delaunay tessellations. Astron. Astrophys. 363, 29–32.

[B62] Schnittger A. Folkers U. Schwab B. Jürgens G. Hülskamp M. (1999). Generation of a spacing pattern: the role of TRIPTYCHON in trichome patterning in Arabidopsis. Plant Cell 11, 1105–1116. 10.1105/tpc.11.6.1105 10368181 PMC144244

[B63] Shapiro M. Schein S. Monasterio F. (1985). Regularity and structure of the spatial pattern of blue cones of macaque retina. J. Am. Stat. Assoc. 80, 803–812 10.1080/01621459.1985.10478185

[B64] Sick S. Reinker S. Timmer J. Schlake T. (2006). WNT and DKK determine hair follicle spacing through a reaction-diffusion mechanism. Science 314, 1447–1450. 10.1126/science.1130088 17082421

[B65] Smith D. L., Watt W. M. (1986). Distribution of lithocysts, trichomes, hydathodes and stomata in leaves of Pilea cadierei gagnep. & guill. (Urticaceae). Ann. Bot. 58, 155–166.

[B66] Snijder B. Pelkmans L. (2011). Origins of regulated cell-to-cell variability. Nat. Rev. Mol. Cell Biol. 12, 119–125. 10.1038/nrm3044 21224886

[B67] Swain P. S. Longtin A. (2006). Noise in genetic and neural networks. Chaos 16:026101. 10.1063/1.2213613 16822033

[B68] Tanemura M. Honda H. Yoshida A. (1991). Distribution of differentiated cells in a cell sheet under the lateral inhibition rule of differentiation. J. Theor. Biol. 153, 287–300. 10.1016/S0022-5193(05)80571-5 1798334

[B69] Torii K. U. (2012). Two-dimensional spatial patterning in developmental systems. Trends Cell Biol. 22, 438–446. 10.1016/j.tcb.2012.06.002 22789547

[B70] Torquato S. (2002). Random Heterogeneous Materials: Microstructure and Macroscopic Properties, Vol. 16 Torquato, AG: Springer 10.1007/978-1-4757-6355-3

[B71] Wallet F. Dussert C. (1997). Multifactorial comparative study of spatial point pattern analysis methods. J. Theor. Biol. 187, 437–447. 10.1006/jtbi.1997.0445 9245582

[B72] Ward M. Wei J. (2002). The existence and stability of asymmetric spike patterns for the schnakenberg model. Stud. Appl. Math. 109, 229–264 10.1111/1467-9590.00223

[B73] Wilkinson D. J. (2009). Stochastic modelling for quantitative description of heterogeneous biological systems. Nat. Rev. Genet. 10, 122–133. 10.1038/nrg2509 19139763

[B74] Woolley T. Baker R. Gaffney E. Maini P. (2011). Stochastic reaction and diffusion on growing domains: understanding the breakdown of robust pattern formation. Phys. Rev. E 84:046216. 10.1103/PhysRevE.84.046216 22181254

